# Spectrum of neurodegenerative disorders in neurology outpatient department: A cross‐sectional study

**DOI:** 10.1002/hsr2.2189

**Published:** 2024-06-12

**Authors:** Aadesh Rayamajhi, Saurav Agrawal, Sabin Acharya, Susmin Karki, Rajeev Ojha

**Affiliations:** ^1^ Maharajgunj Medical Campus Tribhuvan University, Institute of Medicine Kathmandu Nepal; ^2^ Department of Neurology Tribhuvan University Teaching Hospital Kathmandu Nepal

**Keywords:** cross‐sectional study, neurodegenerative disorder, neuroimaging, Nepal, parkinsonian disorder

## Abstract

**Background and Aims:**

Neurodegenerative disorders (NDDs) are a growing global health concern with a rise in prevalence with the aging population, leading to increased healthcare utilization and costs. Understanding its prevalence is crucial for effective diagnostics and resource allocation, especially in developing nations with limited resources. This study aims to explore the frequency and types of NDDs, while also collecting demographic, clinical, and neuro‐radiological data from patients with NDDs attending a tertiary care hospital in Nepal.

**Methods:**

This was a single‐center based cross‐sectional descriptive study conducted at a Neurology outpatient department in a tertiary level hospital in Nepal in which patients aged 18 and above diagnosed with NDDs (May 2023–July 2023) were included. Data were collected and analyzed in SPSS Inc. This study has been presented by the STROBES guidelines.

**Results:**

The mean age of the 71 patients included in the study was 65.6 ± 13.3 years. Parkinsonian disorder (*n* = 41, 57.7%) was the most common NDD diagnosed. Patients belonging to the age group 60–79 years represented 62% of all outpatient visits. Tremors of the upper extremity and impairment of memory were the most commonly encountered first symptoms at onset. Predominant cognitive changes in our study were memory impairment and mood changes. Extrapyramidal features such as gait disturbance, resting tremor, rigidity, and bradykinesia were present. More than half of the patients had age‐related cerebral atrophy on neuroimaging followed by chronic small vessel ischemic changes.

**Conclusion:**

Diagnosing NDDs poses challenges, and our study underscores Parkinsonian disorder, specifically Parkinson's disease, as the prevailing neurodegenerative condition in our population. Emphasizing its prevalence among the elderly, particularly with tremors as the primary presenting symptom, highlights the necessity for targeted interventions in this demographic.

## 1 INTRODUCTION

Neurodegenerative disorders (NDDs) are a group of neurological conditions with diverse clinical and pathological characteristics, affecting specific subgroups of neurons in distinct functional and anatomical regions and progressing relentlessly without a clear explanation for their cause.^1^ All NDDs share a hallmark feature of neurodegeneration which is characterized by the continuous loss of functional neurons, synaptic dysfunction, and the accumulation of modified proteins in the brain, leading to clinical symptoms.^2^, ^3^, ^4^ NDDs can manifest at any point in a human's lifespan with some forms, emerging during infancy, while the majority of them surfacing much later in adulthood usually after the age of 30.^5^

More than 50 million people worldwide are affected by NDDs which presents a significant medical challenge in the modern world as there are no treatments available at present to halt or decelerate the neurodegenerative process.^6^ In global surveys, the yearly occurrence rates of NDDs are typically approximated to range from 10 to 15 cases per 100,000 individuals, with approximately 2% of these cases occurring in individuals aged 65 years and older.^7^ Alzheimer's disease (AD), Parkinson's disease (PD), prion disease, amyotrophic lateral sclerosis (ALS), Huntington's disease (HD), spinal muscular atrophy (SMA), and spinocerebellar ataxia (SCA) are among the prevailing NDDs.^3^ NDDs, such as AD and PD, are a growing global health concern and their prevalence is expected to rise with the aging population, leading to increased healthcare utilization and costs. Despite significant advancements in neurology, managing these disorders remains challenging, given the lack of known cures and limited treatment options. These disorders can significantly impact the quality of life of affected individuals and their families.^8^ Thus, comprehending the range of neurodegenerative disorders is crucial for improving the management and care of these conditions.

There is a lack of studies addressing the various types of NDDs observed in our outpatient setting. Utilizing epidemiological information on frequently occurring diseases within the local community can assist in directing diagnostic tests, ensuring the efficient allocation of limited resources. Consequently, this study aims to determine the frequency and types of NDDs, as well as gather demographic, and clinical information about patients with NDDs visiting a tertiary care hospital in Nepal and help raise awareness amongst healthcare professionals to improve their diagnostic accuracy, leading to earlier and more effective interventions. Additionally, by shedding light on the epidemiological landscape of NDDs in Nepal, our study fills a significant gap in the literature regarding neurological health in this specific geographical context. Moreover, the identification of demographic trends and clinical profiles of NDD patients serves as a foundation for future research and targeted public health initiatives aimed at improving neurological care in Nepal.

## 2 METHODOLOGY

### 2.1 Study design

This was a single‐center based cross‐sectional descriptive study that was carried out in the neurology outpatient department of Tribhuvan University Teaching Hospital, the country's largest referral center. The duration of the study spanned from May 2023 to July 2023 over 3 months, during which a total of 71 patients with (NDDs) were identified. Patients initially screened by physicians and general practitioners for possible NDDs were referred to the neurology clinic. These patients were evaluated by a team of neurologists and subsequently followed up with appropriate investigations and neuroimaging. The patients were diagnosed following the International Classification of Disease‐11 codes for neurology. This study has been reported in line with the STROBES guidelines.^9^

### 2.2 Data collection

The study included patients aged 18 and above diagnosed with a neurodegenerative disorder who had complete medical records and provided informed consent for research, utilizing a convenience sampling method. Information regarding the demographic and disease profile of the patients fulfilling the inclusion criteria was gathered through a structured questionnaire. The questionnaire included four parts: (1) informed consent; (2) demographic information such as age, sex, occupation, education, socioeconomic status, place of residence and lifestyle characteristics and comorbidities (3) clinical information including type of NDD, age and first symptom at onset of disease, pattern of healthcare utilizations (determined by outpatient visits, hospitalization and progression of symptoms), progression and duration of disease, extrapyramidal features and cognitive changes; (4) neuroimaging findings. Study participants whose diagnoses were uncertain throughout the study and those with other neurological disorders that would have confounded the neurodegenerative assessment were excluded from the study.

### 2.3 Ethical consideration

The study received ethical approval from the Institutional Review Committee at Tribhuvan University, Institute of Medicine [Reference number: 522(6‐11) (2079/080)]. This study conformed to the provisions of the Declaration of Helsinki.

### 2.4 Statistical analysis

We conducted data analysis using Statistical Package for Social Sciences ‐ 26 (IBM). We employed descriptive statistics such as frequencies, percentages, means, medians, and standard deviations to depict the distribution and types of disorders observed in the outpatient department.

## 3 RESULTS

The total number of patients enrolled in this study was 71. The mean age of the study participants was 65.6 ± 13.3 and the median age was 67 years. Two‐thirds of patients (*n* = 47, 66.2%) were male. Regarding the occupation of the patients, 21.1% of cases were farmers and 17% of patients owned some form of business. Most of the patients (*n* = 29, 40%) had education up to primary level followed by 26.8% of patients who had completed their secondary education. More than two‐thirds (*n* = 48, 67.6%) of patients belonged to rural areas and 32.4% of patients were from urban areas. Of the total cases, 38% and 33.8% indulged in smoking and drinking habits respectively. A quarter of patients complained of involvement of the bowel and bladder while sleep impairment was noted in 21.1% of patients. The majority of patients (*n* = 31, 43.7%,) had concomitant hypertension and 18.3% of patients had diabetes mellitus (Table 1).

**Table 1:** An overview of demographic and lifestyle characteristics and comorbidities of the study population.

Variables	Frequency (Percentage)
**Age (years)**	
Mean ± standard deviation	65.6 ± 13.3
**Gender**	
Male	47 (66.2)
Female	24 (33.8)
**Occupation**	
Business	12 (16.9)
Farmer	15 (21.1)
Housewife	12 (16.9)
Unable to work	22 (30.9)
Others	10 (14.1)
**Education level**	
Illiterate	8 (11.3)
Primary	29 (40.8)
Secondary	19 (26.8)
Higher Secondary	9 (12.7)
Graduate or above	6 (8.5)
**Place of residence**	
Rural	48 (67.6)
Urban	23 (32.4)
**Social class**	
Lower‐middle class	56 (78.9)
High class	15 (21.1)
**Smoking habit**	
Smoker	27 (38.0)
Nonsmoker	44 (62.0)
**Alcohol consumption**	
Present	24 (33.8)
Absent	47 (66.2)
**Comorbidities**	
Hypertension	31 (43.7)
Diabetes mellitus	13 (18.3)
Cardiovascular disease	6 (8.5)
Thyroid disorder	5 (7.0)

**Figure 1:**
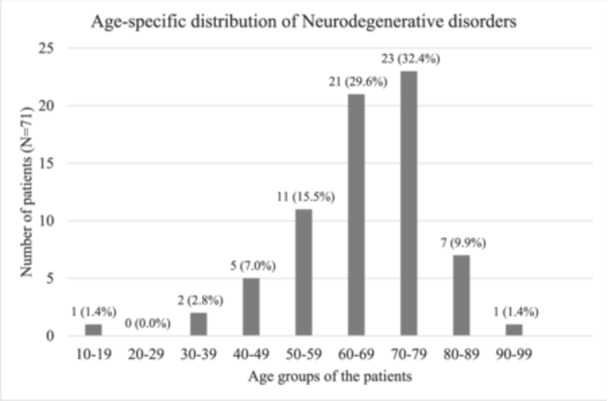
Age‐specific distribution of neurodegenerative disorders (*N* = 71).

The diseases clustered around the age group in the range of 50–79 years accounting for 77.4% of total cases. Approximately, a third of the cases (*n* = 23, *N* = 71) were contributed by patients within the age group of 70‐79 years. This was followed by patients aged 60–69 (*n* = 21) and 50–59 (*n* = 11). The youngest patient in our study was 19 years of age (Figure 1).

**Figure 2:**
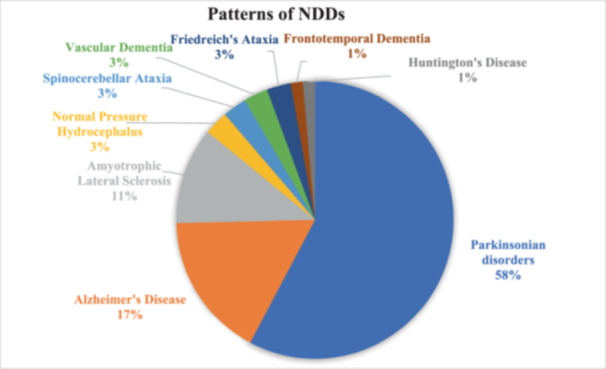
Patterns of neurodegenerative disorders.

**Figure 3:**
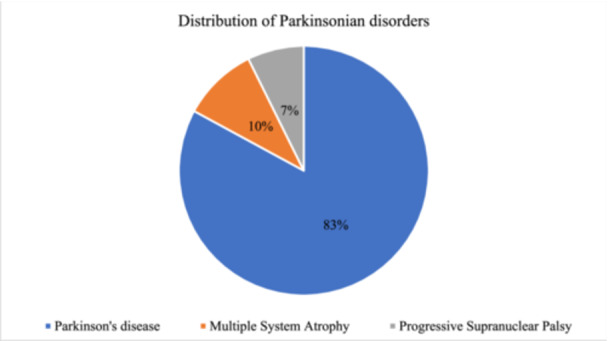
Distribution of Parkinsonian disorder (*n* = 41).

The most prevalent disease in our study was Parkinsonian disorder (*n* = 41, 57.7%) which was followed by AD (*n* = 14, 19.7%) and ALS (*n* = 8, 11.3%). Only a single case of HD and FTD was observed during the study period (Figure 2). Amongst Parkinsonian disorders, PD was noted in 82.9% of cases (*n* = 34, *N* = 41). MSA and PSP contributed 9.7% and 7.3% of total Parkinsonian disorders respectively (Figure 3).

**Figure 4:**
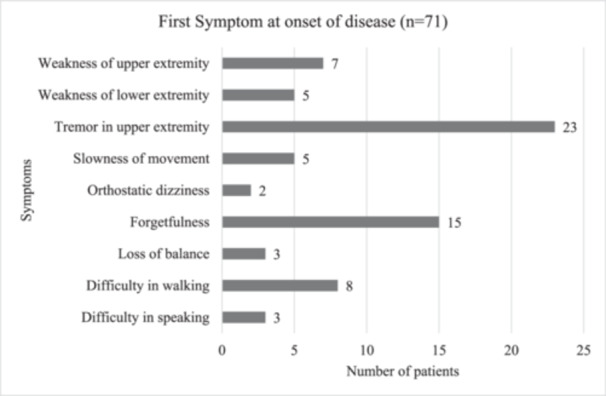
First symptom at the onset of disease.

**Figure 5:**
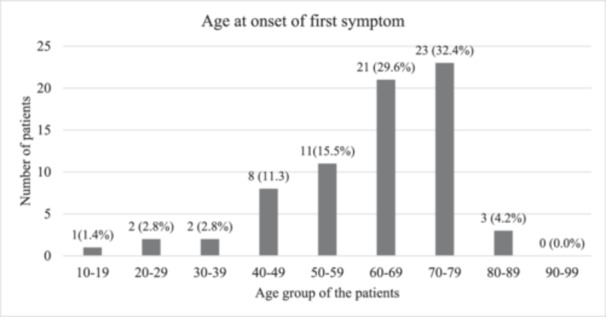
Age at onset of first symptom.

Around a third of the study participants reported tremors in the upper extremities as the first symptom at the onset of the disease (Figure 4). The mean age at onset of the first symptom was 3.8 years. Most patients had their first symptom after the age of 40 years. Only 11.2% of patients were below the age of 50 years when they first noticed their symptoms. Approximately one‐third of patients (*n* = 23, 32.4%) who encountered their initial symptoms of the disease belonged to the 70–79 age group. Additionally, 21 individuals aged 60–69 experienced the onset of their first symptoms (Figure 5).

Higher mental function evaluations of the study participants revealed memory impairment (*n* = 29, 40.8%) as the most common cognitive change. Mood and behavioral changes were seen in 26 cases and speech disturbance was noted in 25 patients (35.2%). Also, 16 patients had changes in their personality.

Extrapyramidal manifestations were observed in most patients. More than half of the patients (*n* = 42, 59.2%) had gait disturbances. Resting tremor was noted in 45.1% of total patients, action tremor in one‐third of the patients while 21.1% of patients had a postural tremor. Bradykinesia was present in more than one‐third of the patients (Table 2).

**Table 2:** Extrapyramidal features on neurological evaluation.

Extrapyramidal features	Number of patients (*n*)	Percentage (%)
Gait disturbance	42	59.2
Ataxia	16	22.5
Rigidity	30	42.3
Tremor		
Resting	32	45.1
Action	25	35.2
Postural	15	21.1
Chorea	1	1.4
Bradykinesia	29	40.8

Among the patients, 36.6% underwent a slow and steady progression over an extended period, spanning years. In contrast, 53.5% demonstrated a swifter disease advancement within a concise 6‐month timeframe. Additionally, 9.9% experienced a rapid disease progression over a relatively brief period, specifically within a month (Table 3). The majority (50.7%) reported symptoms for 0–2 years, while significant portions reported symptoms persisting for 3–5 years (35.2%) and over 5 years (14.1%) (Table 4).

**Table 3:** Duration and progression of disease.

Variables	Number of patients (n)	Percentage (%)
**Duration of symptoms (in years)**		
0–2	36	50.7
3–5	25	35.2
>5	10	14.1
**Progression of disease**		
Slow (over years)	26	36.6
Moderate (over 6 months)	38	53.5
Fast (over a month)	7	9.9

**Table 4:** Pattern of healthcare utilization.

Variables	Number of patients (*n*)	Percentage (%)
**Number of hospitalizations (in a year)**		
0	28	39.4
1	31	43.7
≥2	12	16.9
**Number of outpatient visits (in a year)**		
1–3	27	38
4–6	34	47.9
7–9	5	7
≥10	5	7
**Number of emergency room visits (in a year)**		
0	63	88.7
1	8	11.3

Most patients had some kind of neuroimaging changes and around a quarter of patients had normal findings in magnetic resonance imaging. More than half of the patients had age‐related cerebral atrophy. The next common finding observed was white matter high T2 signal intensities suggestive of chronic small vessel ischemic changes (Table 5).

**Table 5:** Neuroimaging changes.

Neuroimaging findings	Number of patients (*n*)	Percentage (%)
Age‐related cerebral atrophy	38	53.5
White matter high T2 signal (chronic small vessel ischemic changes)	33	46.5
T2 signal hyper‐intensities in the bilateral corticospinal tract and deep white matter	3	4.2
Hippocampal, para‐hippocampal gyrus, and medial temporal lobe atrophy	2	2.8
Ventricular enlargement disproportionate to cerebral atrophy	2	2.8
Marked atrophy of dorsal midbrain especially of anteroposterior diameter	2	2.8
Frontotemporal atrophy	1	1.4
Normal MRI finding	18	25.4

## 4 DISCUSSION

As life expectancy continues to rise, the incidence of NDDs is on the rise in both developed and developing nations. Understanding the prevalence and specific patterns of these diseases within local communities is crucial for guiding diagnostic efforts and ensuring the effective allocation of limited resources, particularly in developing countries. Although there is ample data on NDDs in developed nations, there is a paucity of information regarding the epidemiology and clinical manifestations of these disorders in developing countries. This study aims to explore the frequency and types of NDDs, while also collecting demographic, clinical, and neuroradiological data from patients with NDDs attending a tertiary care hospital in Nepal.

Age stands out as the single most contributing risk factor for the onset of various NDDs although growing evidence suggests a person's susceptibility can be equally influenced by a combination of their genetic makeup and environmental factors.^3^ In our study, the mean age of study participants was 65.6 years (standard deviation 13.3), and the mean age at the onset of disease was 61.7 years. The age group between 60 and 79 years represented 62% of all outpatient visits. A review of NDDs by Hou et. al revealed similar conclusions along with the rising prevalence of disease with increasing age of 65 years.^10^ Among the study participants, a majority of patients, comprising 66.2% of the total sample (*n* = 47), were male. The review articles by Hou et. Al., and Lekoubou et. Al., disclose NDDs such as AD to be more common amongst females while PD and LBD amongst males.^10^, ^11^ The predominance of males in our study can be attributed to the fact that the majority of participants exhibited Parkinsonian disorder.

In our center, Parkinsonian disorder surpassed AD as the predominant NDD, contrary to existing literature. The discrepancy may stem from factors such as AD patients' lack of awareness, reluctance to seek treatment, and communal living in joint families where caregivers influence treatment decisions. AD, often considered physiological, sees home‐based remedies. Additionally, neuropsychiatrists manage dementia patients in our center. In contrast, Parkinsonian disorder, with its treatable motor symptoms and cognitive clarity, leads to increased treatment‐seeking behavior and a larger patient population in our center's outpatient department.

Predominant cognitive changes in our study were memory impairment, and mood changes followed by speech disturbance and personality changes. Extrapyramidal features such as gait disturbance, resting tremors, rigidity, and bradykinesia were present. A prospective cross‐sectional study from a tertiary care center in India on 102 patients of PD reported the majority with bradykinesia followed by resting tremor, rigidity, postural instability, and cognitive impairment.^12^ Predominant non‐motor symptoms in our study were bowel and bladder involvement followed by sleep disturbance and depression as was seen in a cross‐sectional study done in a tertiary care center in Pakistan.^13^, ^14^

A meta‐analysis has revealed an elevated risk of Parkinson's disease associated with environmental factors, such as residing in rural areas, using well water, engaging in farming, and exposure to pesticides.^15^ Our study, where over two‐thirds of patients came from rural areas and 21.1% reported farming as their primary income source, aligns with these findings. This higher representation from rural areas may also be linked to the scarcity of skilled manpower and resources in those regions, with limited tertiary care centers like ours. The urban‐rural healthcare resource disparity is evident, contributing to the observed pattern of increased neurodegenerative disorder cases in rural regions. Moreover, our study identified comorbidities like hypertension, type 2 diabetes mellitus, cardiac disorders, and thyroid disorders as associated with NDDs, emphasizing the multifaceted nature of factors influencing these disorders.^16^, ^17^, ^18^

NDDs are often underrecognized, undertreated, and under‐managed in low‐income countries. The debilitating nature of neurodegeneration establishes it as a predominant contributor to global dependence and disability.^19^ A study assessing the burden of NDDs in the Eastern Mediterranean region (EMR) demonstrated that these disorders constituted 5.4% of the overall Disability‐Adjusted Life Years and 4.6% of the total Years Lived with Disability within the aging population of the EMR.^20^ Our study identifies varied disease progression patterns among patients, with a substantial group showing slow progression over years and half experiencing moderate progression within 6 months. This diversity suggests distinct patient phenotypes, warranting personalized treatments. The duration of symptoms also varied, emphasizing the heterogeneous nature of the disease duration within the studied population. The data on healthcare utilization patterns shed light on the impact of the disease on the healthcare system. Throughout this time frame, less than half of the patients (47.9%) had outpatient department visits four to six times a year, and 43.7% had at least one hospitalization. Overall, these findings provide valuable insights for healthcare professionals in tailoring treatment strategies and resource allocation based on the varying needs of the patient population.

A study by Breen et. Al. showed that the time from motor symptom onset to seeing a primary care physician was significantly longer than the time from that visit to a Parkinson's disease diagnosis.^21^ Diagnosing NDDs is challenging due to diverse clinical presentations and overlapping causes. Neuroimaging, crucial for evaluating and monitoring brain changes, holds promise for accurate diagnosis.^22^ In our study, the majority of patients exhibited neuroimaging alterations, with around 25% having normal results in magnetic resonance imaging. Over half showed age‐related cerebral atrophy, while AD‐specific changes were observed in only two patients. Although only two patients were diagnosed with vascular dementia, in fact, more patients demonstrated white matter hyperintensities which might be just clinically silent age‐related changes.^23^

Our study findings emphasize the need for heightened awareness of NDDs, particularly among older adults who often present with symptoms after the age of 40. Targeted screening protocols, especially for common initial symptoms like tremors in the upper extremities, are crucial for early diagnosis and intervention. Given the high prevalence of Parkinsonian disorders, including PD, specialized care facilities are essential to manage their complex symptoms effectively. Additionally, understanding disease progression patterns is vital for allocating resources for timely intervention and support services, improving patient outcomes. Considering the prevalence of comorbidities such as hypertension and diabetes mellitus, integrated care approaches addressing both neurological and systemic health needs are imperative.

The 3‐month duration of our study may have limited its ability to capture the long‐term dynamics of neurodegenerative disorders. Challenges in transportation and geographical remoteness in Nepal hindered patient follow‐up. Cultural and language variations affected effective communication, potentially impacting symptom reporting accuracy. Additionally, our reliance on convenience sampling for quick patient recruitment may introduce bias by favoring easily accessible participants, limiting findings' generalizability across populations or settings. The unavailability of advanced diagnostic equipment such as function MRI and PET scans, economic constraints, and socioeconomic disparities influenced the precision of assessments and access to tests. Despite facing limitations including manpower constraints, our study provides valuable insights within its defined scope.

## 5 CONCLUSION

This study unveils a diverse spectrum of neurodegenerative disorders within our population, with Parkinsonian disorder emerging as the predominant one, with the majority of cases attributed to PD. This disorder exhibited a heightened prevalence among the elderly population, where tremors were the primary presenting symptom for most patients. Notably, cognitive changes and additional extrapyramidal symptoms were also documented. Further research at the community level is necessary to elucidate the observed variation, as our study revealed a lower prevalence of AD compared to Parkinsonian disorder. Common neuroimaging findings in our study encompassed age‐related cerebral atrophy and the presence of hyperintense T2 signal intensities in white matter.

## AUTHOR CONTRIBUTIONS

**Aadesh Rayamajhi**: Conceptualization; methodology; software; writing–original draft; writing–review and editing; data curation; investigation; validation; formal analysis; supervision; funding acquisition; visualization; project administration; resources. **Saurav Agrawal**: Conceptualization; methodology; software; data curation; investigation; validation; formal analysis; supervision; funding acquisition; visualization; project administration; resources; writing–original draft; writing–review & editing. **Sabin Acharya**: Data curation; writing–review and editing. **Susmin Karki**: Data curation. **Rajeev Ojha**: Conceptualization; methodology; formal analysis; writing–review and editing; supervision.

## CONFLICT OF INTEREST STATEMENT

The authors declare no conflict of interest.

## ETHICS STATEMENT

The study was approved by the Institutional Review Committee of Tribhuvan University, Institute of Medicine [Reference proposal number: 522(6‐11) (2079/080)].

## TRANSPARENCY STATEMENT

The lead author Aadesh Rayamajhi affirms that this manuscript is an honest, accurate, and transparent account of the study being reported; that no important aspects of the study have been omitted; and that any discrepancies from the study as planned (and, if relevant, registered) have been explained.

## Data Availability

The datasets analyzed during the current study are provided within the manuscript. The major data file used during the current study is available in supplementary file as “research data final. doc”.
