# Correction to “Spectrum of neurodegenerative disorders in neurology outpatient department: A cross‐sectional study”

**DOI:** 10.1002/hsr2.2249

**Published:** 2024-07-10

Rayamajhi A, Agrawal S, Acharya S, Karki S, Ojha R. Spectrum of neurodegenerative disorders in neurology outpatient department: a cross‐sectional study. *Health Sci Rep*. 2024;7:e2189. doi:10.1002/hsr2.2189

During the article submission process, I, Aadesh Rayamajhi, the first and submitting author, mistakenly listed myself as corresponding author. The author responsibilities were predefined and another co‐author, Dr. Rajeev Ojha, was originally designated for this role. The correct corresponding author is Dr. Rajeev Ojha.

We apologize for this error.

